# Prognostic Value of the Uric Acid-to-Albumin Ratio in Patients Undergoing Successful Percutaneous Coronary Intervention for Chronic Total Occlusion

**DOI:** 10.3390/jcdd13060282

**Published:** 2026-06-22

**Authors:** Qiheng Wan, Song Wen, Jiquan Xiao, Feihuang Han, Zehan Huang, Dunliang Ma, Feng Wang, Yuqing Huang, Bin Zhang

**Affiliations:** 1 Guangdong Cardiovascular Institute, Guangdong Provincial People’s Hospital, Guangdong Academy of Medical Sciences, Guangzhou 510080, China; 13662056294@163.com (Q.W.); xiaojiquan888@163.com (J.X.); han_feihuang@163.com (F.H.); zehanhuang@foxmail.com (Z.H.); drmadunliang@163.com (D.M.); wfeng7830@163.com (F.W.); hyq513@126.com (Y.H.); 2 Department of Cardiology, Fuwai Hospital, National Center for Cardiovascular Diseases, Chinese Academy of Medical Sciences and Peking Union Medical College, Beijing 100037, China; drsongwen@163.com

**Keywords:** chronic total occlusion, percutaneous coronary intervention, uric acid-to-albumin ratio, prognosis

## Abstract

Introduction: The uric acid-to-albumin ratio (UAR) is a novel cardiovascular biomarker, but its prognostic value in patients undergoing percutaneous coronary intervention (PCI) for chronic total occlusion (CTO) remains unknown. Materials and Methods: This retrospective study enrolled 1513 consecutive patients who underwent successful CTO-PCI at a single center from February 2011 to December 2023. Patients were stratified by baseline UAR tertiles. The primary endpoint was major adverse cardiovascular and cerebrovascular events (MACCE), and the secondary endpoint was all-cause mortality. Multivariable Cox regression and restricted cubic spline (RCS) analyses were performed. Results: During a median follow-up of 810 days, patients in the highest UAR tertile had significantly higher rates of MACCE (18.5%, 10.1%, and 7.5% across tertiles; *p* < 0.001) and all-cause mortality (10.7%, 3.8%, and 2.0%; *p* < 0.001). After multivariable adjustment, each one-unit increase in UAR was associated with a 6% higher risk of MACCE (HR 1.06; 95% CI 1.02–1.10; *p* = 0.002) and a 9% higher risk of all-cause mortality (HR 1.09; 95% CI 1.04–1.14; *p* < 0.001). Patients in the highest UAR tertile had significantly increased risks of MACCE (HR 1.90; 95% CI 1.25–2.90; *p* = 0.003) and all-cause mortality (HR 3.40; 95% CI 1.62–7.12; *p* = 0.001) compared with those in the lowest UAR tertile. RCS analysis showed significant overall associations between UAR and both MACCE and all-cause mortality, with no significant evidence of nonlinearity. Conclusions: Elevated baseline UAR was independently associated with long-term MACCE and all-cause mortality after successful CTO-PCI. These findings support UAR as a readily available prognostic marker but do not establish causality or support UAR-guided therapeutic decision-making. Prospective studies are needed for validation.

## 1. Introduction

Globally, atherosclerotic coronary heart disease persists as a primary cause of cardiovascular morbidity and mortality, leading to significant long-term disability and premature death [1]. The incidence of coronary artery disease (CAD) is progressively increasing, fueled by pervasive shifts in lifestyle habits and an aging demographic, which consequently places a severe and growing strain on healthcare systems worldwide. Defined as an absolute antegrade Thrombolysis in Myocardial Infarction (TIMI) grade 0 flow lasting for a minimum of three months, coronary chronic total occlusion (CTO) constitutes the most advanced anatomical presentation of progressive atherosclerosis [2]. Contemporary studies have reported that CTO is present in roughly 16 to 20 percent of individuals diagnosed with CAD [3]. A significant decrease in regional myocardial blood flow is caused by CTO, which leads to either reversible ischemia or irreversible infarction, ultimately resulting in diminished quality of life and a poor long-term outlook for patients. Notwithstanding progressive advancements in percutaneous coronary intervention (PCI), the revascularization of CTOs remains hindered by less-than-ideal success rates during procedures, a high susceptibility to restenosis, and variable long-term patient results [4,5,6].

As the ultimate byproduct of purine metabolism, serum uric acid (SUA) has been suggested to play a role in both the onset and progression of atherosclerosis through various harmful pathways. These include endothelial impairment [7], vascular smooth muscle cell proliferation, intensification of oxidative stress, production of free radicals and an increased tendency toward thrombosis [8]. Hyperuricemia is thought to accelerate atherosclerotic progression chiefly through endothelial dysfunction, which stands as the central pathway among these deleterious mechanisms. Translational evidence substantiating this link comes from observations that individuals with elevated SUA possess markedly higher circulating concentrations of established endothelial dysfunction biomarkers, namely albuminuria and plasma endothelin-1, directly associating hyperuricemia with overt vascular injury [9]. In contrast, albumin serves as a classic example of a negative acute-phase reactant. Low levels of albumin in the blood have been consistently associated with unfavorable cardiovascular profiles [10]. Recent evidence suggests that the uric acid-to-albumin ratio (UAR) represents a novel biomarker that captures the complementary pathophysiological information provided by its individual components. Findings from recent cohort and case–control investigations indicate that UAR demonstrates a superior and incremental predictive value for incident cardiovascular events compared to either SUA or albumin measured alone [11,12].

The objective of this study was to evaluate whether the UAR serves as an independent predictor of long-term clinical outcomes following CTO-PCI. To our knowledge, this is the first investigation specifically designed to assess the prognostic significance of UAR within this particular procedural setting.

## 2. Materials and Methods

### 2.1. Study Population

A retrospective, single-center cohort analysis was conducted on consecutive patients who underwent CTO-PCI at Guangdong Provincial People’s Hospital between February 2011 and December 2023. Revascularization was pursued in patients presenting with angina refractory to optimal medical therapy and/or objective evidence of ischemia or viable myocardium within the territory supplied by the CTO. Assessment of ischemia primarily involved non-invasive stress modalities, including stress echocardiography, myocardial perfusion imaging, or cardiac magnetic resonance. For regions exhibiting severe dysfunction, myocardial viability was evaluated using cardiac magnetic resonance, dobutamine stress echocardiography, or single-photon emission computed tomography (SPECT). In cases where non-invasive findings were inconclusive, selective utilization of fractional flow reserve (FFR) measurements was employed.

**Figure 1:**
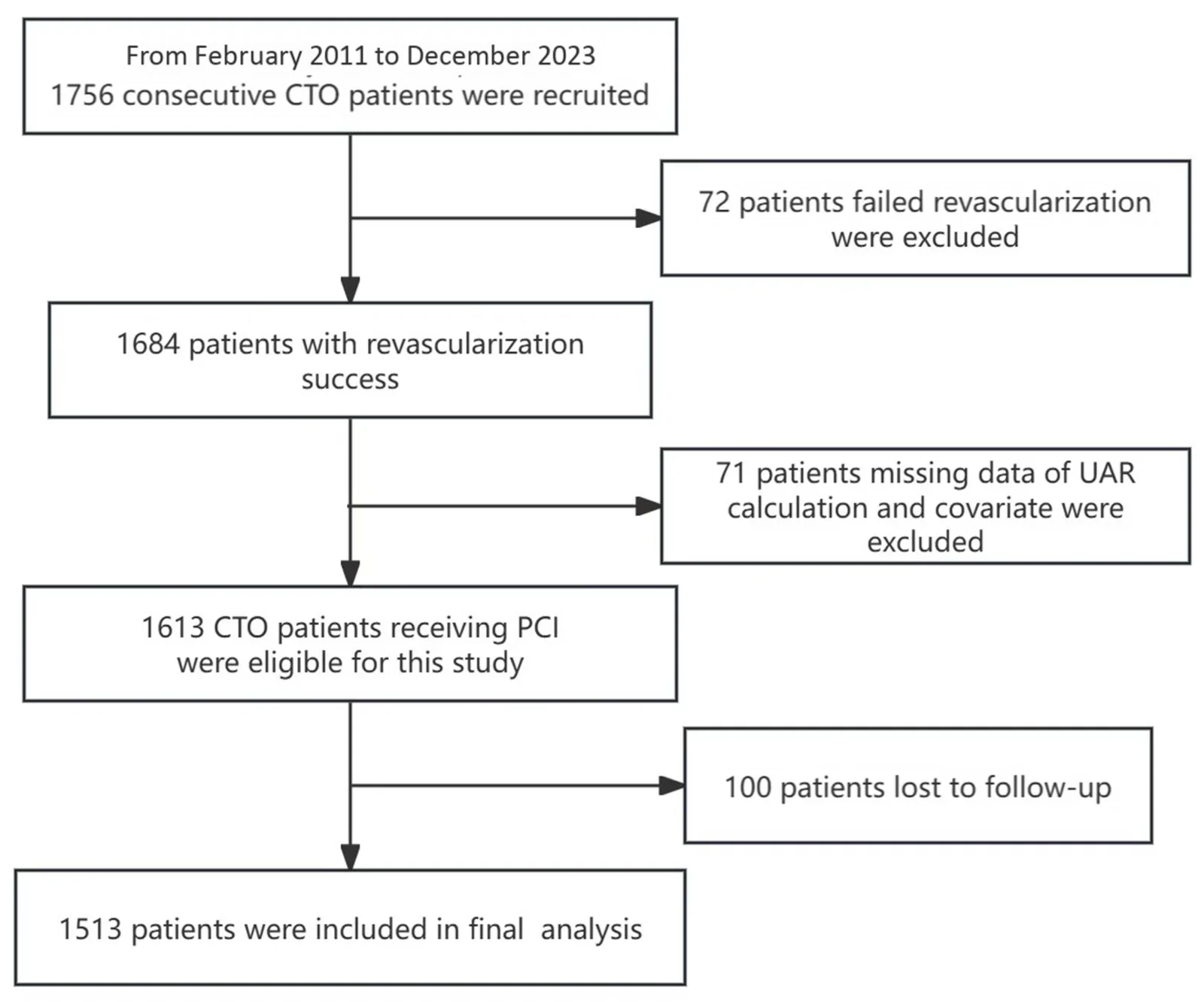
Study Flowchart. From February 2011 to December 2023, a total of 1756 consecutive patients with chronic total occlusion (CTO) were recruited. After excluding 72 patients with failed revascularization, 71 patients with missing data for UAR calculation or covariates, and 100 patients lost to follow-up, 1513 patients were included in the final analysis.

This study enrolled eligible adults (≥18 years) presenting with angiographically confirmed coronary CTO who had provided written informed consent for data collection. After removing 72 cases of procedural failure, 100 patients lost to follow-up (overall follow-up loss rate ~5.7%), and 71 subjects with incomplete UAR or essential covariate information, a total of 1513 individuals with successful CTO revascularization formed the basis of the final analysis (Figure 1). Baseline characteristics, procedural data, and long-term outcomes were compared between 3 groups according to the tertiles of UAR. Approval for the research protocol was obtained from the Institutional Ethics Committee at Guangdong Provincial People’s Hospital, and all procedures complied with the ethical standards set forth in the Declaration of Helsinki.

### 2.2. Clinical Definitions and Measurements

The primary outcome measure for the study was a composite of major adverse cardiovascular and cerebrovascular events (MACCE)—including all-cause death, non-fatal myocardial infarction (MI), stroke, and target vessel revascularization (TVR)—during follow-up. Mortality from any cause constituted the secondary endpoint. Cardiovascular death was defined as death due to cardiovascular causes, including acute MI, sudden cardiovascular death, heart failure, stroke, cardiovascular procedures, cardiovascular hemorrhage, and other cardiovascular etiologies [13]. MI was diagnosed according to the Society for Cardiovascular Angiography and Interventions (SCAI) criteria [14]. TVR was defined as any clinically indicated repeat revascularization—whether via PCI or coronary artery bypass grafting (CABG)—involving the original target vessel.

The procedural techniques for retrograde CTO-PCI have already been thoroughly documented in an earlier publication [15]. All coronary angiographic and PCI procedures were conducted using either femoral or radial arterial access, or a combination of both. The operating physicians determined the interventional strategies, which encompassed the choice of crossing techniques, guiding catheters, stent types, and the application of intravascular imaging. Each operator possessed extensive experience as a high-volume interventionalist, having completed more than 300 lifetime CTO-PCI procedures and maintaining a minimum of 50 annual cases in the capacity of primary operator.

A CTO was characterized by an angiographically confirmed total obstruction of antegrade flow (TIMI grade 0), with the occlusion estimated to have persisted for at least three months [2]. The duration of occlusion was ascertained through clinical history, taking into account factors such as the initiation of anginal symptoms, history of prior myocardial infarction, or comparison with earlier coronary angiograms. All laboratory parameters including SUA and albumin for UAR calculation were routinely tested at baseline before the PCI procedure. UAR was calculated as the ratio of SUA concentration (μmol/L) to serum albumin concentration (g/L).

According to most published studies, technical success for CTO-PCI is characterized by the establishment of antegrade flow of at least TIMI grade 2 in every distal branch with a diameter of 2.5 mm or greater, along with less than 30% residual stenosis in the target lesion, at the conclusion of the intervention [16]. The following periprocedural adverse events were documented: in-hospital death, non-fatal myocardial infarction, coronary perforation, cardiac tamponade, stroke, and emergency cardiac surgery.

### 2.3. Follow-Up

The median follow-up duration was 810 days (interquartile range: 409 to 1230 days). The monitoring of clinical events involved checking hospital readmission records, communicating directly with referring physicians, and conducting outpatient clinical evaluations. All reported MACCE underwent additional verification by physicians through a systematic evaluation of the source clinical documentation.

### 2.4. Statistical Analyses

For continuous variables, data presentation depended on their distribution: mean ± standard deviation (SD) was used for normally distributed data, while median with interquartile range (IQR) was employed for non-normally distributed data, with normality assessed via the Kolmogorov–Smirnov test. Categorical variables were expressed as frequencies and percentages. Group comparisons were conducted utilizing the unpaired Student’s *t*-test for normally distributed continuous variables, the Kruskal–Wallis test for non-normally distributed continuous variables, the Pearson chi-square test for categorical variables, and Fisher’s exact test in cases where expected cell counts were low. Estimation of cumulative event rates was performed using the Kaplan–Meier method, with comparisons made via the log-rank test. To evaluate the association between UAR and the risks of MACCE and all-cause mortality, univariable and multivariable Cox proportional hazards regression analyses were undertaken, yielding hazard ratios (HRs) accompanied by 95% confidence intervals (CIs). Covariates in the multivariable models were selected based on clinical relevance and previous PCI/CTO-PCI prognostic literature. The Schoenfeld residual test (cox.zph) was applied to verify the proportional hazards (PH) assumption for each endpoint of MACCE, including all-cause death, non-fatal MI, stroke, and TVR. Examination of potential nonlinear relationships between continuous UAR and outcomes involved the integration of restricted cubic splines (RCS) within the Cox models. Assessment of consistency across predefined clinical strata was accomplished through subgroup analyses and interaction testing. All statistical computations were carried out using R software (version 4.3.1), with a two-sided *p*-value below 0.05 considered indicative of statistical significance.

## 3. Results

### 3.1. Entire Population Findings

The median UAR in the overall cohort was 10.48 (IQR: 8.69–12.41). Based on tertile distribution, patients were stratified into three groups: T1 (Low, n = 505), T2 (Medium, n = 505), and T3 (High, n = 503). A summary of baseline clinical characteristics and medication use is provided in Table 1. The cohort had a median age of 59 years (IQR: 52.00, 67.00), with 91.6% being male. Diabetes mellitus was present in 39.8% of patients, while 59.6% had a diagnosis of hypertension.

Across increasing UAR tertiles, patients were more likely to be male and had progressively lower eGFR and LVEF. Patients in the highest UAR tertile also had a higher prevalence of dyslipidemia and higher levels of CRP, LDL, BNP, troponin T, BUN, D-dimer, SUA, ApoB100, and LDH, whereas albumin and ApoAI levels were lower. Diabetes mellitus was more frequent in the lowest UAR tertile. No significant differences were observed among the three groups in hypertension, smoking status, prior MI, prior stroke, prior PCI, or prior CABG.

With respect to prior medication use, SGLT2 inhibitor use was more frequent in the lowest UAR tertile, whereas the use of statins, β-blockers, DAPT, calcium channel blockers, and ACEI/ARB was similar across the three groups. The distribution of target CTO arteries and the prevalence of multivessel disease were also comparable among UAR tertiles.

### 3.2. Clinical Outcomes

A progressive increase in clinical event rates was observed across ascending UAR tertiles. The incidence of MACCE increased from 7.5% in T1 to 10.1% in T2 and 18.5% in T3 (*p* < 0.001). Similarly, the rates of all-cause mortality (2.0%, 3.8%, and 10.7%; *p* < 0.001) and cardiovascular death (0.8%, 1.8%, and 8.0%; *p* < 0.001) increased across UAR tertiles. No significant differences were observed among the three groups in the incidence of non-fatal MI, stroke, or TVR.

**Figure 2:**
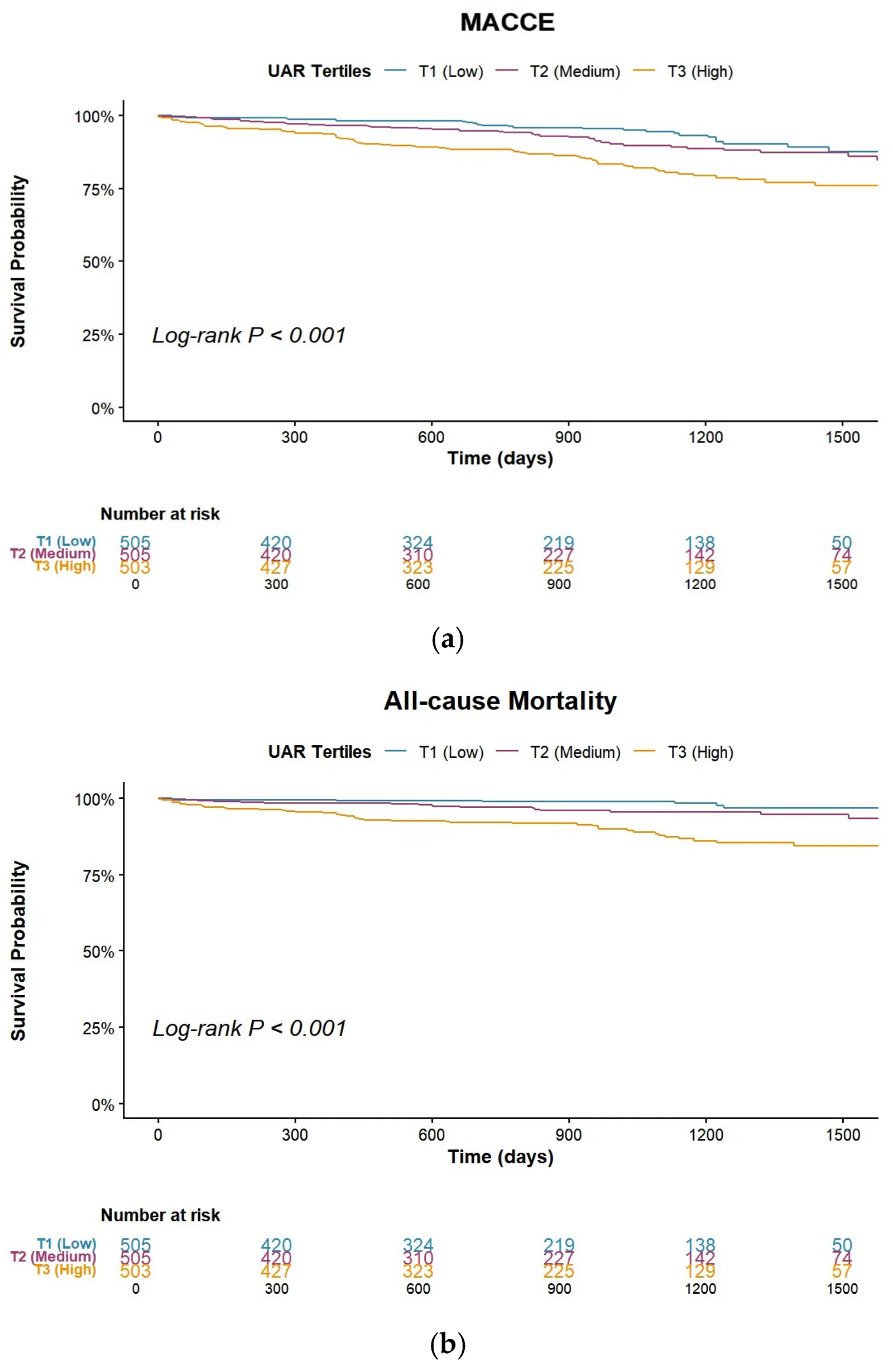
Kaplan–Meier curves for time to MACCE (**a**) and time to all-cause mortality (**b**) in patients undergoing successful CTO-PCI, stratified by tertiles of the uric acid-to-albumin ratio (UAR).

Kaplan–Meier analysis showed that patients in the highest UAR tertile had the lowest MACCE-free survival and overall survival during follow-up. The log-rank test showed significant differences among the three UAR tertiles for both MACCE-free survival and overall survival (both *p* < 0.001) (Figure 2).

Univariate Cox proportional hazards analysis revealed that patients in the highest UAR tertile (T3) had higher risks of all-cause mortality (HR: 5.27; 95% CI: 2.68–10.35; *p* < 0.001) and MACCE (HR: 2.27; 95% CI: 1.56–3.32; *p* < 0.001) relative to those in the lowest tertile (T1). After multivariable adjustment, the highest UAR tertile remained significantly associated with increased risks of both MACCE and all-cause mortality in Models 2 and 3 (Table 2). In the fully adjusted Model 3, each one-unit increase in UAR was associated with a 6% higher risk of MACCE (HR 1.06; 95% CI 1.02–1.10; *p* = 0.002) and a 9% higher risk of all-cause mortality (HR 1.09; 95% CI 1.04–1.14; *p* < 0.001). Compared with patients in the lowest UAR tertile, those in the highest tertile had a 1.90-fold higher risk of MACCE (95% CI 1.25–2.90; *p* = 0.003) and a 3.40-fold higher risk of all-cause mortality (95% CI 1.62–7.12; *p* = 0.001) in the fully adjusted model.

For individual components of MACCE, the Schoenfeld residual test indicated a significant violation of the PH assumption (all *p* < 0.05). Notably, the significant association between UAR and composite MACCE was predominantly driven by all-cause mortality, and UAR had no meaningful predictive effect on non-fatal MI, stroke or TVR. To address potential over-adjustment caused by the coexistence of multiple inflammatory biomarkers sharing overlapping pathophysiological pathways, we performed a sensitivity analysis by excluding C-reactive protein (CRP) from the covariates in Model 3 (Supplementary Table S1). After excluding CRP, the independent associations between elevated UAR and MACCE as well as all-cause mortality remained statistically significant.

Additionally, the results of the subgroup analyses for MACCE and all-cause mortality are presented in Table 3 and Table 4, respectively. For MACCE (Table 3), a higher UAR level was also associated with a higher risk of worse outcome in similar subgroups: age > 60 years, males, non-diabetic patients, eGFR ≤ 60 mL/min, and smokers. A significant interaction was found for eGFR (*p* for interaction = 0.024), suggesting that the association between UAR and MACCE was stronger in patients with impaired renal function. For all-cause mortality (Table 4), a higher UAR level was associated with a significantly increased risk in subgroups including patients aged > 60 years, males, those without diabetes mellitus, those with eGFR ≤ 60 mL/min, smokers, and those without dyslipidemia. A significant interaction was observed for age (*p* for interaction = 0.032), indicating that the prognostic impact of UAR was more pronounced in older patients.

**Figure 3:**
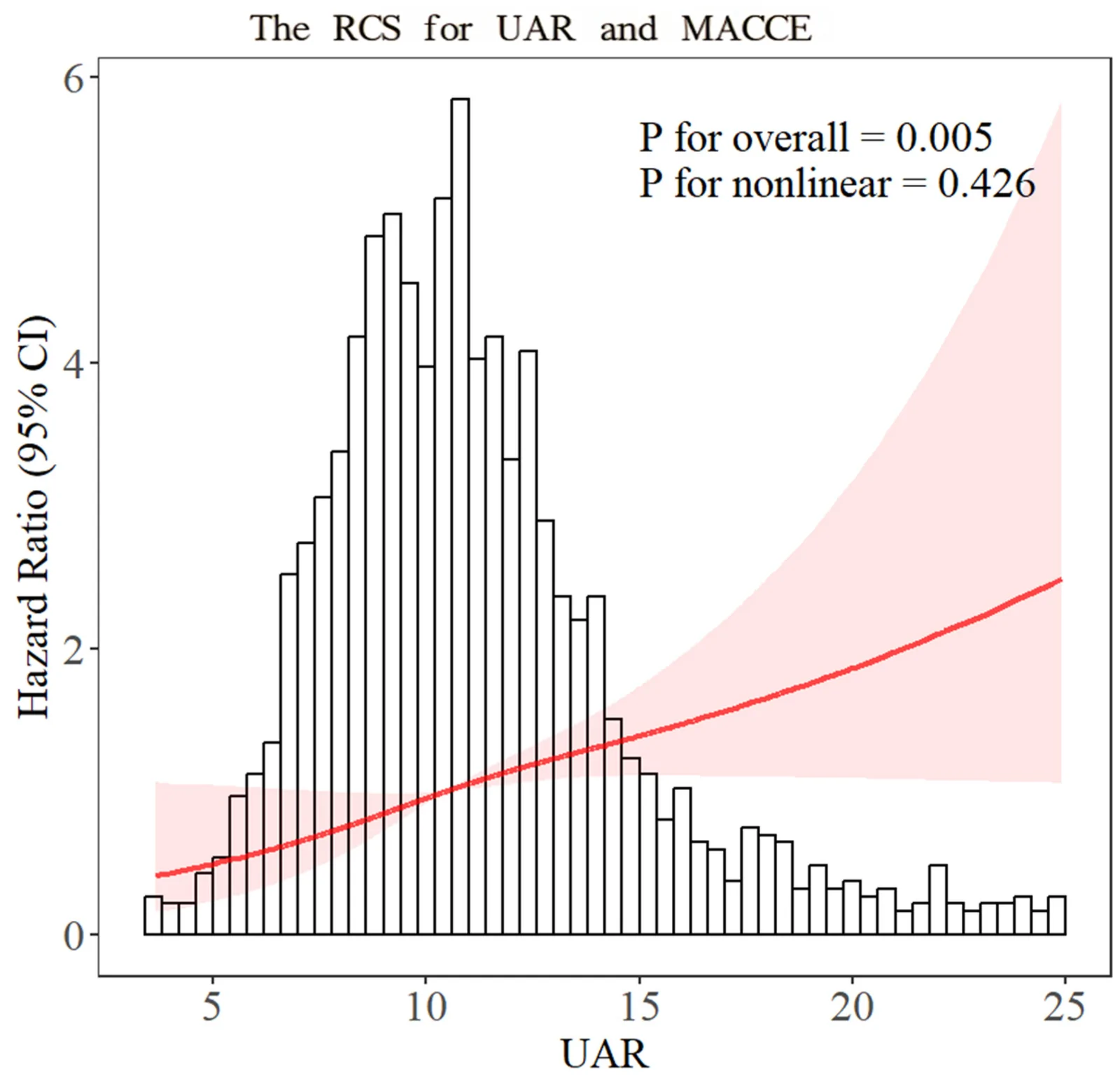
Multivariable-adjusted hazard ratios (solid line) and 95% confidence intervals (shaded area) for major adverse cardiovascular and cerebrovascular events (MACCE) according to continuous UAR levels. The histogram shows the distribution of UAR. The model was adjusted for potential confounders. The association between UAR and MACCE was statistically significant overall (*p* for overall = 0.005), with no significant evidence of nonlinearity (*p* for nonlinearity = 0.426), suggesting an approximately linear increase in risk with higher UAR levels.

**Figure 4:**
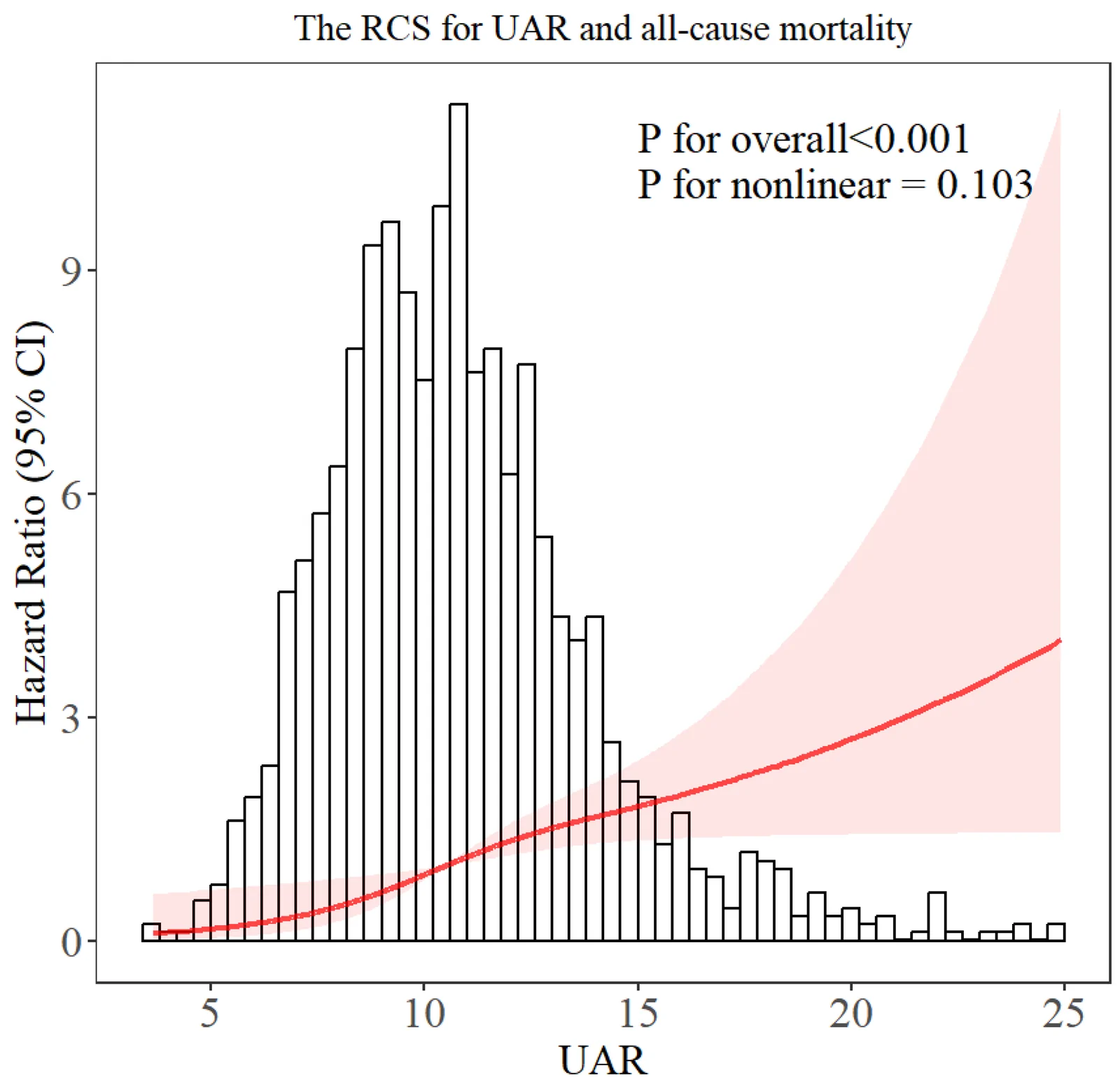
Multivariable-adjusted hazard ratios (solid line) and 95% confidence intervals (shaded area) for all-cause mortality according to continuous UAR levels. The histogram shows the distribution of UAR. The model was adjusted for potential confounders. The association between UAR and all-cause mortality was statistically significant overall (*p* for overall < 0.001), with no significant evidence of nonlinearity (*p* for nonlinearity = 0.103), suggesting an approximately linear increase in risk with higher UAR levels.

These findings suggest that each unit increase in UAR may be associated with a higher risk of adverse outcomes in clinically vulnerable subgroups, particularly older patients (>60 years) and those with impaired renal function (eGFR ≤ 60 mL/min). RCS analyses using multivariable-adjusted Cox models (Model 3) showed significant overall associations between UAR and both MACCE (*p* for overall = 0.005) and all-cause mortality (*p* for overall < 0.001). No significant evidence of nonlinearity was observed for either MACCE (*p* for nonlinearity = 0.426) or all-cause mortality (*p* for nonlinearity = 0.103), suggesting approximately linear increases in risk with higher UAR levels across the observed range (Figure 3 and Figure 4).

## 4. Discussion

In this single-center retrospective cohort of patients who underwent successful PCI for CTO, a higher baseline UAR was associated with an increased risk of long-term MACCE and all-cause mortality. This association remained significant after adjustment for major clinical and laboratory covariates. Importantly, the association was also preserved in a sensitivity analysis in which CRP was removed from the fully adjusted model, suggesting that the prognostic relationship between UAR and adverse outcomes was not solely explained by adjustment for systemic inflammation. Patients in the highest UAR tertile had lower eGFR and LVEF and higher levels of BNP, troponin T, BUN, D-dimer, and SUA, together with lower albumin levels, suggesting that UAR may integrate several domains of vulnerability, including renal dysfunction, impaired cardiac reserve, systemic inflammation, oxidative stress, nutritional status, and thrombotic tendency. Therefore, our findings should be interpreted as suggesting a potential association between elevated UAR and worse prognosis after successful CTO-PCI, rather than establishing a causal relationship or a treatment-guiding biomarker. Confirmation in larger prospective, multicenter cohorts is required.

Previous studies have evaluated UAR in different coronary populations. UAR has been investigated in chronic coronary syndromes [17], NSTEMI [18], and broader acute coronary syndrome populations [19], supporting its relevance as a composite marker related to adverse inflammatory, nutritional, and metabolic status in coronary artery disease. In PCI-treated patients, UAR has also been associated with post-procedural outcomes, including in-stent restenosis [20] and long-term cardiovascular death [11]. Our study extends these observations to patients with CTO who underwent successful revascularization and suggests that UAR may retain prognostic information in this complex PCI population. However, the present findings also differ from studies focused on lesion-specific outcomes. In our cohort, elevated UAR was associated with all-cause mortality, cardiovascular death, and MACCE, whereas no significant differences were observed for nonfatal MI, stroke, or TVR. This pattern suggests that UAR may primarily reflect systemic or global cardiovascular risk rather than a CTO-specific mechanism.

The biological plausibility of this association is supported by the complementary information provided by the two components of UAR. Uric acid is the final product of purine metabolism and has been linked to endothelial dysfunction, oxidative stress, inflammation, vascular smooth muscle activation, and adverse renal and cardiovascular phenotypes [21,22,23]. Xanthine oxidase activity, which contributes to uric acid generation, is also an important source of reactive oxygen species. Experimental and clinical studies have shown that inhibition of this pathway can improve endothelial function and reduce oxidative stress markers, although evidence for hard cardiovascular benefits remains inconsistent and population-dependent [24,25,26]. Thus, elevated uric acid in high-risk cardiovascular populations may be less a direct therapeutic target than a marker of increased oxidative and metabolic stress.

Albumin, in contrast, has antioxidant, anti-inflammatory, antiplatelet, and anticoagulant properties and is also a marker of nutritional and inflammatory status [27,28]. Lower albumin levels have been associated with adverse outcomes in coronary heart disease. In a large cohort of patients with coronary heart disease, serum albumin showed a linear inverse association with both all-cause and cardiovascular mortality [29]. A comprehensive meta-analysis including more than 2 million patients further showed that hypoalbuminemia was associated with higher risks of venous thromboembolism, MI, and stroke [27]. These findings support the concept that low albumin may identify patients with a systemic proinflammatory, prothrombotic, and nutritionally vulnerable phenotype. In this context, UAR may provide a compact marker that combines the adverse information of higher uric acid with the loss of albumin-associated protective effects. This concept is supported by recent evidence on composite inflammation–nutrition indices in coronary intervention populations. Trimarchi et al. reported that the Advanced Lung Cancer Inflammation Index, which combines serum albumin, body mass index, and the neutrophil-to-lymphocyte ratio, independently predicted all-cause mortality in STEMI patients undergoing primary PCI and showed better discrimination than the neutrophil-to-lymphocyte ratio alone [30]. This supports the rationale for integrating inflammatory and nutritional parameters into a single prognostic index. Because CRP may lie on the same inflammatory pathway reflected in part by UAR, adjustment for CRP could potentially attenuate the observed association through over-adjustment. The persistence of the association after removing CRP from the fully adjusted model supports the robustness of our findings and suggests that UAR may capture prognostic information beyond a single inflammatory marker.

The baseline profile of the high-UAR group further supports this interpretation. Patients in the highest UAR tertile had lower LVEF and higher BNP and troponin T levels, indicating a greater burden of myocardial injury and impaired cardiac reserve. They also had lower eGFR and higher BUN, suggesting worse renal function, which may both increase uric acid levels and amplify cardiovascular risk. The stronger association between UAR and MACCE among patients with eGFR ≤ 60 mL/min/1.73 m^2^ is consistent with this interaction between renal dysfunction and cardiovascular vulnerability. In addition, the higher prevalence of dyslipidemia and higher ApoB100 levels in the high-UAR group suggest that UAR may coexist with a more atherogenic metabolic profile. These findings do not prove that UAR directly contributes to adverse outcomes, but they suggest that elevated UAR identifies a phenotype characterized by overlapping renal, metabolic, inflammatory, and myocardial risk.

The RCS analyses further characterized the dose–response relationship between UAR and adverse outcomes. In the multivariable-adjusted models, UAR showed significant overall associations with both MACCE and all-cause mortality, while no significant evidence of nonlinearity was observed for either endpoint. These findings suggest that the risk of adverse outcomes increased gradually with higher UAR levels across the observed range, rather than showing a distinct threshold effect. This pattern is consistent with the Cox regression analyses, in which UAR was associated with higher risks of both MACCE and all-cause mortality when modeled as a continuous variable and when categorized into tertiles. The gradual increase in risk may reflect the cumulative systemic risk burden captured by UAR, including renal dysfunction, impaired cardiac reserve, inflammation, oxidative stress, and nutritional status. Notably, the confidence intervals widened at the upper range of UAR, likely because fewer patients had very high UAR values; therefore, estimates in this range should be interpreted cautiously. Overall, the spline findings support UAR as a continuous risk-associated marker rather than a clinically actionable cutoff for guiding treatment decisions.

The absence of significant differences in nonfatal MI and TVR is clinically important. If UAR were primarily a marker of CTO lesion-related vulnerability or restenosis, stronger associations with recurrent MI or repeat target-vessel revascularization might have been expected. Instead, the association was most apparent for mortality and the composite endpoint. This finding supports the possibility that UAR reflects the overall biological risk of the patient rather than the behavior of the successfully treated CTO segment. Accordingly, elevated UAR may indicate a subgroup of patients with a higher overall risk profile after CTO-PCI. In such patients, UAR may provide additional prognostic context alongside conventional clinical assessment, but it should not be used alone to guide treatment decisions. However, no prospective study has tested whether UAR-guided intensification of treatment improves outcomes in CTO-PCI patients. Clinical recommendations based on UAR should therefore remain cautious.

Taken together, our findings suggest that UAR is a readily available biomarker associated with long-term adverse outcomes after successful CTO-PCI. Its value may lie not in identifying a CTO-specific pathway, but in capturing the combined burden of oxidative stress, inflammation, renal dysfunction, impaired cardiac reserve, and reduced albumin-associated protective capacity. Future studies should evaluate whether UAR improves established risk models beyond conventional clinical, angiographic, and procedural variables and whether changes in UAR over time provide additional prognostic information.

This study has several limitations. First, this was a single-center retrospective observational study, and residual confounding or selection bias cannot be excluded. Loss to follow-up and exclusions due to missing data may also have introduced selection bias or informative censoring if these patients differed systematically from those included in the final analysis. In addition, MACCE and cardiovascular death included heterogeneous events such as stroke and cardiovascular hemorrhage, which may not be directly related to the CTO lesion or the CTO-PCI procedure itself. Therefore, these findings should be interpreted as reflecting overall cardiovascular risk rather than CTO-specific or target-vessel-related risk. Second, UAR was calculated only from baseline SUA and albumin measurements; serial biomarker data were unavailable, so we could not assess whether longitudinal changes in UAR were associated with outcomes. Third, the long inclusion period may have introduced temporal bias, as CTO-PCI techniques, operator experience, device availability, lesion complexity, and background medical therapy may have changed over time. In particular, increasing operator experience may have allowed more complex CTO lesions to be attempted in later years. Finally, this study included only patients with successful CTO-PCI, and the findings may not be generalizable to patients with failed CTO-PCI, medical therapy alone, or surgical revascularization.

## 5. Conclusions

In this single-center retrospective cohort of patients undergoing successful PCI for CTO, elevated baseline UAR was associated with higher risks of long-term MACCE and all-cause mortality. UAR may reflect a broader systemic risk profile involving inflammation, oxidative stress, renal dysfunction, impaired cardiac reserve, and reduced albumin-associated protective capacity. However, these findings should be interpreted cautiously and do not establish causality or support UAR-guided therapeutic decision-making. Prospective multicenter studies are needed to validate the prognostic value of UAR, determine whether it improves established risk prediction models, and assess whether UAR-informed management can improve clinical outcomes.

## Figures and Tables

**Table 1:** Characteristics of patients according to UAR tertiles.

Variables	All n = 1513	T1 (Low) n = 505	T2 (Medium) n = 505	T3 (High) n = 503	*p*-Value
Demographics and clinical characteristics					
Age, (years)	59.00 (52.00, 67.00)	60.00 (53.00, 68.00)	59.00 (52.00, 66.00)	59.00 (51.00, 66.00)	0.076
Male, (%)	1386.0 (91.6%)	448.0 (88.7%)	464.0 (91.9%)	474.0 (94.2%)	0.007
SBP, (mmHg)	130.00 (119.00, 143.00)	131.00 (120.00, 143.00)	131.00 (121.00, 143.00)	128.00 (115.00, 142.00)	0.012
DBP, (mmHg)	78.00 (70.00, 85.00)	78.00 (70.00, 85.00)	78.00 (70.00, 85.00)	78.00 (69.00, 86.00)	0.7
Heart rate, (beats/min)	74.00 (67.00, 82.00)	74.00 (66.00, 81.00)	74.00 (68.00, 81.00)	74.00 (67.00, 84.00)	0.6
eGFR, (mL/min)	85.37 (68.03, 97.40)	90.64 (79.10, 100.33)	86.85 (71.30, 98.01)	72.34 (56.54, 92.30)	<0.001
LVEF, (%)	58.00 (46.00, 63.00)	59.00 (53.00, 64.00)	60.00 (49.00, 64.00)	53.00 (40.00, 62.00)	<0.001
Comorbidities					
HTN, n, (%)	902.0 (59.6%)	290.0 (57.4%)	302.0 (59.8%)	310.0 (61.6%)	0.4
DM, (%)	602.0 (39.8%)	227.0 (45.0%)	188.0 (37.2%)	187.0 (37.2%)	0.015
Dyslipidemia, n (%)	423.0 (28.0%)	106.0 (21.0%)	152.0 (30.1%)	165.0 (32.8%)	<0.001
Smoker, n (%)	588.0 (38.9%)	180.0 (35.6%)	200.0 (39.6%)	208.0 (41.4%)	0.2
Prior MI, n (%)	237.0 (15.7%)	68.0 (13.5%)	84.0 (16.6%)	85.0 (16.9%)	0.2
Prior CABG, n (%)	36.0 (2.4%)	10.0 (2.0%)	18.0 (3.6%)	8.0 (1.6%)	0.093
Prior Stroke, n (%)	75.0 (5.0%)	24.0 (4.8%)	23.0 (4.6%)	28.0 (5.6%)	0.7
Prior PCI, n (%)	757.0 (50.0%)	252.0 (49.9%)	239.0 (47.3%)	266.0 (52.9%)	0.2
Laboratory measurements					
Hb, (g/L)	135.00 (125.00, 146.00)	136.00 (126.00, 146.00)	136.00 (126.00, 145.00)	134.00 (121.00, 145.00)	0.038
WBC, (10^9^/L)	6.96 (5.84, 8.25)	6.79 (5.76, 8.03)	6.87 (5.70, 8.14)	7.19 (6.06, 8.54)	<0.001
PLT, (10^9^/L)	217.00 (180.00, 263.00)	212.00 (176.00, 259.00)	216.00 (180.00, 258.00)	221.00 (185.00, 271.00)	0.056
ALT, (U/L)	22.00 (16.00, 32.00)	22.00 (16.00, 32.00)	22.00 (16.00, 31.00)	22.00 (15.00, 32.00)	0.7
CRP, (mg/L)	1.97 (0.50, 6.20)	1.50 (0.50, 5.30)	2.23 (0.50, 5.97)	2.40 (0.50, 7.92)	<0.001
BNP, (pg/mL)	229.40 (73.40, 749.70)	137.80 (58.50, 456.80)	185.90 (63.00, 595.90)	488.40 (136.30, 1434.00)	<0.001
TNT, (pg/mL)	17.20 (9.70, 41.24)	13.00 (8.20, 23.10)	17.90 (9.60, 40.30)	24.30 (12.60, 63.80)	<0.001
Lp(a), (mg/L)	197.00 (75.00, 461.00)	186.00 (68.00, 390.00)	197.00 (77.00, 502.00)	221.00 (82.60, 487.00)	0.14
LDL, (mmol/L)	2.39 (1.87, 2.95)	2.24 (1.75, 2.77)	2.35 (1.85, 2.88)	2.59 (2.10, 3.29)	<0.001
BUN, (mmol/L)	5.59 (4.52, 6.83)	5.32 (4.44, 6.33)	5.40 (4.42, 6.59)	6.20 (4.89, 8.16)	<0.001
TBIL, (μmol/L)	11.70 (9.20, 14.90)	11.90 (9.50, 15.30)	11.90 (9.40, 15.10)	11.20 (8.90, 14.10)	0.001
FIB, (g/L)	3.51 (3.07, 4.18)	3.40 (3.03, 3.96)	3.45 (3.03, 4.02)	3.72 (3.18, 4.52)	<0.001
CK, (U/L)	87.00 (63.00, 122.00)	85.00 (63.00, 115.00)	89.00 (65.00, 125.00)	86.00 (60.00, 125.00)	0.2
CK-MB, (U/L)	10.20 (10.00, 13.00)	10.20 (10.00, 13.00)	10.30 (10.00, 13.10)	10.30 (10.00, 13.20)	>0.9
AST, (U/L)	22.00 (18.00, 27.00)	21.00 (17.00, 27.00)	22.00 (18.00, 27.00)	22.00 (18.00, 27.00)	0.9
ApoAI, (g/L)	1.11 (1.01, 1.24)	1.14 (1.03, 1.26)	1.12 (1.02, 1.26)	1.10 (0.97, 1.20)	<0.001
ApoB100, (g/L)	0.75 (0.60, 0.91)	0.70 (0.56, 0.85)	0.75 (0.59, 0.90)	0.80 (0.65, 0.97)	<0.001
LDH, (U/L)	159.00 (139.00, 183.00)	157.00 (137.00, 179.00)	158.00 (138.00, 180.00)	164.00 (141.00, 191.00)	0.007
D-dimer, (mg/L)	340.00 (240.00, 597.00)	330.00 (220.00, 550.00)	300.00 (230.00, 530.00)	420.00 (270.00, 760.00)	<0.001
ALB, (g/L)	38.80 (36.56, 41.00)	39.50 (37.60, 42.20)	38.92 (36.93, 41.00)	37.80 (35.30, 39.94)	<0.001
Scr, (μmol/L)	84.00 (72.90, 100.19)	77.70 (67.19, 88.22)	84.00 (72.60, 96.50)	97.00 (78.36, 116.92)	<0.001
SUA, (μmol/L)	404.90 (337.00, 478.00)	314.20 (277.70, 343.90)	408.05 (382.30, 434.60)	519.50 (468.55, 572.00)	<0.001
NEUT, (%)	0.60 (0.54, 0.66)	0.60 (0.54, 0.66)	0.60 (0.54, 0.66)	0.60 (0.56, 0.66)	0.7
UAR	10.48 (8.69, 12.41)	8.09 (7.14, 8.69)	10.49 (9.90, 11.01)	13.47 (12.41, 15.26)	<0.001
Prior medication use					
SGLT2i, n (%)	272.0 (18.0%)	126.0 (25.0%)	80.0 (15.9%)	66.0 (13.1%)	<0.001
Statin, n (%)	1494.0 (98.7%)	497.0 (98.4%)	500.0 (99.0%)	497.0 (98.8%)	0.7
β-Blocker, n (%)	1211.0 (80.0%)	406.0 (80.4%)	405.0 (80.2%)	400.0 (79.5%)	>0.9
DAPT, n (%)	1477.0 (97.6%)	494.0 (97.8%)	496.0 (98.2%)	487.0 (96.8%)	0.3
CCB, n (%)	318.0 (21.0%)	100.0 (19.8%)	118.0 (23.4%)	100.0 (19.9%)	0.3
ACEI/ARB, n (%)	902.0 (59.7%)	292.0 (57.8%)	306.0 (60.7%)	304.0 (60.4%)	0.6
Target CTO artery					
RCA, n (%)	683.0 (45.1%)	217.0 (43.0%)	226.0 (44.8%)	240.0 (47.7%)	
RCA + LAD, n (%)	98.0 (6.5%)	35.0 (6.9%)	26.0 (5.1%)	37.0 (7.4%)	
RCA + LAD + LCX, n (%)	22.0 (1.5%)	10.0 (2.0%)	7.0 (1.4%)	5.0 (1.0%)	
RCA + LCX, n (%)	55.0 (3.6%)	16.0 (3.2%)	14.0 (2.8%)	25.0 (5.0%)	
LAD, n (%)	530.0 (35.0%)	189.0 (37.4%)	191.0 (37.8%)	150.0 (29.8%)	
LAD + LCX, n (%)	63.0 (4.2%)	19.0 (3.8%)	23.0 (4.6%)	21.0 (4.2%)	
LCX, n (%)	59.0 (3.9%)	19.0 (3.8%)	17.0 (3.4%)	23.0 (4.6%)	
LM, n (%)	3.0 (0.2%)	0.0 (0.0%)	1.0 (0.2%)	2.0 (0.4%)	
Multi-vessel disease, n (%)	1354.0 (89.5%)	448.0 (88.7%)	459.0 (90.9%)	447.0 (88.9%)	0.5
clinical outcome					
MACCE, n (%)	182.0 (12.0%)	38.0 (7.5%)	51.0 (10.1%)	93.0 (18.5%)	<0.001
All-cause death, n (%)	83.0 (5.5%)	10.0 (2.0%)	19.0 (3.8%)	54.0 (10.7%)	<0.001
Cardiovascular death, n (%)	53.0 (3.5%)	4.0 (0.8%)	9.0 (1.8%)	40.0 (8.0%)	<0.001
Nonfatal MI, n (%)	9.0 (0.6%)	1.0 (0.2%)	2.0 (0.4%)	6.0 (1.2%)	0.11
Stroke, n (%)	14.0 (0.9%)	1.0 (0.2%)	7.0 (1.4%)	6.0 (1.2%)	0.10
TVR, n (%)	84.0 (5.6%)	26.0 (5.1%)	26.0 (5.1%)	32.0 (6.4%)	0.6

SBP, Systolic Blood Pressure; DBP, Diastolic Blood Pressure; eGFR, estimated Glomerular Filtration Rate; LVEF, Left Ventricular Ejection Fraction; CRP, C-reactive protein; LDL, low-density lipoprotein cholesterol; Hb, Hemoglobin; WBC, White Blood Cell; PLT, Platelet; ALT, Alanine Aminotransferase; BNP, brain natriuretic peptide; TNT, Troponin T; Lp(a), Lipoprotein(a); BUN, Blood Urea Nitrogen; TBIL, Total Bilirubin; FIB, Fibrinogen; CK, Creatine Kinase; CK-MB, Creatine Kinase-MB; AST, Aspartate Aminotransferase; ApoAI, Apolipoprotein AI; ApoB100, Apolipoprotein B100; LDH, Lactate Dehydrogenase; D-Dimer, D-Dimer; ALB, Albumin; Scr, Serum Creatinine; SUA, Serum Uric Acid; NEUT, Neutrophil Ratio; UAR, Uric Acid-to-Albumin Ratio; SGLT2i, Sodium-Glucose Cotransporter-2 Inhibitor; MACCE, Major Adverse Cardiovascular Events; Nonfatal MI, Non-fatal Myocardial Infarction; TVR, Target Vessel Revascularization; HTN, Hypertension; DM, Diabetes Mellitus; Prior MI, Prior Myocardial Infarction; Prior CABG, Prior Coronary Artery Bypass Grafting; Prior PCI, Prior Percutaneous Coronary Intervention; RCA, Right Coronary Artery; LAD, Left Anterior Descending Artery; LCX, Left Circumflex Artery; LM, Left Main Coronary Artery; Multi, Multi-vessel; DAPT, Dual Antiplatelet Therapy; CCB, Calcium Channel Blocker; ACEI, Angiotensin-Converting Enzyme Inhibitor; ARB, Angiotensin II Receptor Blocker.

**Table 2:** Associations between UAR and MACCE and all-cause mortality in participants with CTO.

	Model 1		Model 2		Model 3	
	HR (95% CI)	*p*-Value	HR (95% CI)	*p*-Value	HR (95% CI)	*p*-Value
MACCE						
UAR (Per −1 unit)	1.11 (1.07–1.15)	<0.0001	1.11 (1.07–1.15)	<0.0001	1.06 (1.02–1.10)	0.002
UAR (Tertiles)						
Low	Reference		Reference		Reference	
Medium	1.18 (0.78–1.80)	0.433	1.24 (0.81–1.89)	0.325	1.16 (0.75–1.78)	0.505
High	2.27 (1.56–3.32)	<0.001	2.36 (1.61–3.45)	<0.001	1.90 (1.25–2.90)	0.003
*p* for trend		<0.001		<0.001		0.015
All-cause mortality						
UAR (Per −1 unit)	1.18 (1.14–1.22)	<0.0001	1.18 (1.14–1.23)	<0.0001	1.09 (1.04–1.14)	<0.001
UAR (Tertiles)						
Low	Reference		Reference		Reference	
Medium	1.77 (0.82–3.81)	0.146	1.91 (0.88–4.13)	0.099	1.64 (0.75–3.58)	0.214
High	5.27 (2.68–10.35)	<0.001	5.66 (2.88–11.66)	<0.001	3.40 (1.62–7.12)	0.001
*p* for trend		<0.001		<0.001		<0.001

Model 1 adjusts for: None. Model 2 adjusts for: DM, sex, age, HTN, Prior MI, Smoker, Statin, DAPT. Model 3 adjusts for: DM, sex, age, HTN, eGFR, LVEF, Prior MI, Smoker, Multi-vessel disease, Statin, DAPT, LDL, BNP, CRP. eGFR, estimated Glomerular Filtration Rate; LVEF, Left Ventricular Ejection Fraction; CRP, C-reactive protein; LDL, low-density lipoprotein cholesterol; BNP, brain natriuretic peptide; UAR, Uric Acid-to-Albumin Ratio; MACCE, Major Adverse Cardiovascular Events; HTN, Hypertension; DM, Diabetes Mellitus; Prior MI, Prior Myocardial Infarction; DAPT, Dual Antiplatelet Therapy.

**Table 3:** UAR level and risk of MACCE in CTO-PCI patients, stratified by various baseline characteristics.

Variable	Count	Percent	Point Estimate	Lower	Upper	*p* Value	*p* for Interaction
Overall	1513	100	1.09	1.05	1.14	<0.001	
sex							0.91
FEMALE	127	8.4	1.13	0.99	1.28	0.073	
MALE	1386	91.6	1.06	1.02	1.11	0.003	
age							0.06
≤60 y	814	53.8	1.01	0.95	1.08	0.752	
>60 y	699	46.2	1.11	1.05	1.18	<0.001	
LVEF							0.393
>40%	1242	82.1	1.07	1.01	1.14	0.015	
≤40%	271	17.9	1.05	0.99	1.11	0.087	
HTN							0.541
NO	611	40.4	1.06	0.99	1.14	0.117	
YES	902	59.6	1.08	1.02	1.14	0.008	
DM							0.435
NO	911	60.2	1.11	1.04	1.19	0.003	
YES	602	39.8	1.03	0.98	1.09	0.244	
eGFR							0.024
>60	1261	83.3	0.99	0.94	1.05	0.715	
≤60	252	16.7	1.15	1.07	1.24	<0.001	
Smoker							0.705
NO	925	61.1	1.05	1.01	1.1	0.023	
YES	588	38.9	1.12	1.03	1.22	0.006	
Dyslipidemia							0.809
NO	1090	72	1.07	1.03	1.12	0.001	
YES	423	28	1.02	0.92	1.13	0.665	
Prior MI							0.797
NO	1276	84.3	1.06	1.01	1.11	0.01	
YES	237	15.7	1.08	0.98	1.18	0.138	
Prior PCI							0.157
NO	756	50	1.1	1.03	1.18	0.005	
YES	757	50	1.06	1.01	1.12	0.023	

LVEF, Left Ventricular Ejection Fraction; HTN, Hypertension; DM, Diabetes Mellitus; eGFR, estimated Glomerular Filtration Rate; Prior MI, Prior Myocardial Infarction; Prior PCI, Percutaneous Coronary Intervention.

**Table 4:** UAR level and risk of all-cause death in CTO-PCI patients, stratified by various baseline characteristics.

Variable	Count	Percent	Point Estimate	Lower	Upper	*p* Value	*p* for Interaction
Overall	1513	100	1.09	1.05	1.14	<0.001	
sex							0.757
FEMALE	127	8.4	1.21	1.03	1.42	0.022	
MALE	1386	91.6	1.09	1.04	1.14	<0.001	
age							0.032
≤60 y	814	53.8	1.01	0.93	1.09	0.84	
>60 y	699	46.2	1.16	1.09	1.25	<0.001	
LVEF							0.078
>40%	1242	82.1	1.17	1.08	1.27	<0.001	
≤40%	271	17.9	1.06	1	1.13	0.059	
HTN							0.292
NO	611	40.4	1.08	0.99	1.18	0.093	
YES	902	59.6	1.12	1.05	1.2	0.001	
DM							0.55
NO	911	60.2	1.16	1.06	1.28	0.002	
YES	602	39.8	1.06	1	1.12	0.06	
eGFR							0.18
>60	1261	83.3	1	0.92	1.08	0.951	
≤60	252	16.7	1.16	1.07	1.26	<0.001	
Smoker							0.265
NO	925	61.1	1.07	1.01	1.13	0.018	
YES	588	38.9	1.22	1.1	1.36	<0.001	
Dyslipidemia							0.122
NO	1090	72	1.1	1.05	1.16	<0.001	
YES	423	28	0.98	0.82	1.17	0.825	
Prior MI							0.945
NO	1276	84.3	1.09	1.04	1.15	0.001	
YES	237	15.7	1.08	0.96	1.21	0.201	
Prior PCI							0.578
NO	756	50	1.15	1.05	1.25	0.002	
YES	757	50	1.1	1.02	1.17	0.008	

LVEF, Left Ventricular Ejection Fraction; HTN, Hypertension; DM, Diabetes Mellitus; eGFR, estimated Glomerular Filtration Rate; Prior MI, Prior Myocardial Infarction; Prior PCI, Percutaneous Coronary Intervention.

## Data Availability

No material from other sources was reproduced in this manuscript. All content is original. The data presented in this study are available on request from the corresponding author. The data are not publicly available due to privacy and ethical restrictions.

## Supplementary Materials

The following supporting information can be downloaded at: https://www.mdpi.com/article/10.3390/jcdd13060282/s1, Table S1: Associations between UAR and MACCE and all-cause mortality in participants with CTO adjusted for all covariates in Model 3 except C-reactive protein.

## References

[1] Roth G.A., Mensah G.A., Johnson C.O., Addolorato G., Ammirati E., Baddour L.M., Barengo N.C., Beaton A.Z., Benjamin E.J., Benziger C.P. (2020). Global Burden of Cardiovascular Diseases and Risk Factors, 1990–2019. J. Am. Coll. Cardiol..

[2] Dash D. (2018). Coronary chronic total occlusion intervention: A pathophysiological perspective. Indian Heart J..

[3] Azzalini L., Karmpaliotis D., Santiago R., Mashayekhi K., Di Mario C., Rinfret S., Nicholson W.J., Carlino M., Yamane M., Tsuchikane E. (2022). Contemporary Issues in Chronic Total Occlusion Percutaneous Coronary Intervention. JACC Cardiovasc. Interv..

[4] Sanchez-Jimenez E., El-Mokdad R., Chaddad R., Cortese B. (2022). Drug-coated balloon for the management of coronary chronic total occlusions. Rev. Cardiovasc. Med..

[5] Leite L., Campos G., Silva R., Jorge E., Oliveira-Santos M., Gomes A., Gonçalves L., Castelo-Branco M., Abrunhosa A., Ferreira M.J. (2022). The association of collaterals with myocardial ischemia and viability in chronic total occlusions. Int. J. Cardiovasc. Imaging.

[6] Fagu A., Berger T., Pingpoh C., Kondov S., Kreibich M., Minners J., Czerny M., Siepe M. (2023). In-Hospital Outcomes Following Surgical Revascularization of Chronic Total Coronary Occlusions. Medicina.

[7] Diaz M.N., Frei B., Vita J.A., Keaney J.F. (1997). Antioxidants and Atherosclerotic Heart Disease. N. Engl. J. Med..

[8] Anker S.D., Leyva F., Poole-Wilson P.A., Kox W.J., Stevenson J.C., Coats A.J. (1997). Relation between serum uric acid and lower limb blood flow in patients with chronic heart failure. Heart.

[9] Stakhova T.Y., Pulin A.A., Severova M.M., Shovskaya T.N., Minakova E.G., Oleynikova E.B., Lebedeva M.V., Stakhova T.Y., Pulin A.A., Severova M.M. (2011). Clinical implication of endothelial dysfunction in patients with essential arterial hypertension and urate dysbolism with renal damage. Ter. Arkhiv.

[10] Karacali K., Kapansahin T., Oner D.Y., Ilhan B.C., Salman A., Yarlioglu M. (2025). Relationship between C-reactive protein and uric acid to albumin ratio and coronary collateral circulation in patients with chronic total occlusion. Coron. Artery Dis..

[11] Li S., Chen H., Zhou L., Cui H., Liang S., Li H. (2022). The uric acid to albumin ratio: A novel predictor of long-term cardiac mortality in patients with unstable angina pectoris after percutaneous coronary intervention. Scand. J. Clin. Lab. Investig..

[12] Zhang Y., Xu Z., He W., Lin Z., Liu Y., Dai Y., Chen W., Chen W., He W., Duan C. (2022). Elevated Serum Uric Acid/Albumin Ratio as a Predictor of Post-Contrast Acute Kidney Injury After Percutaneous Coronary Intervention in Patients with ST-Segment Elevation Myocardial Infarction. J. Inflamm. Res..

[13] Garcia-Garcia H.M., McFadden E.P., Farb A., Mehran R., Stone G.W., Spertus J., Onuma Y., Morel M.-A., van Es G.-A., Zuckerman B. (2018). Standardized End Point Definitions for Coronary Intervention Trials: The Academic Research Consortium-2 Consensus Document. Circulation.

[14] Moussa I.D., Klein L.W., Shah B., Mehran R., Mack M.J., Brilakis E.S., Reilly J.P., Zoghbi G., Holper E., Stone G.W. (2013). Consideration of a New Definition of Clinically Relevant Myocardial Infarction After Coronary Revascularization. J. Am. Coll. Cardiol..

[15] Megaly M., Xenogiannis I., Abi Rafeh N., Karmpaliotis D., Rinfret S., Yamane M., Burke M.N., Brilakis E.S. (2020). Retrograde Approach to Chronic Total Occlusion Percutaneous Coronary Intervention. Circ. Cardiovasc. Interv..

[16] Zhang D., Nan N., Xue Y., Zhang M., Tian J., Chen C., Zhang M., Song X. (2024). Comparison of successful versus failed percutaneous coronary intervention in patients with chronic total occlusion: A systematic review and meta-analysis. Cardiol. J..

[17] Yalcinkaya D., Karacali K., Ilhan B.C., Yarlioglues M. (2024). Relation Between Serum Uric Acid to Albumin Ratio and Severity of Chronic Coronary Artery Disease. Angiology.

[18] Çakmak E.Ö., Bayam E., Çelik M., Kahyaoğlu M., Eren K., Imanov E., Karagöz A., İzgi İ.A. (2020). Uric Acid-to-Albumin Ratio: A Novel Marker for the Extent of Coronary Artery Disease in Patients with Non-ST-Elevated Myocardial Infarction. Pulse.

[19] Prasad B.K., Dutta B., Iqbal F. (2025). Utility of C-reactive Protein-to-Albumin and Uric Acid-to-Albumin Ratios for Assessing Coronary Artery Disease Severity in Acute Coronary Syndrome: A Study From a Major Tertiary Care Center in Northeast India. Cureus.

[20] Liu W., Ding K., Bao J., Hu Y., Gui Y., Ye L., Wang L. (2023). Relationship between uric acid to albumin ratio and in-stent restenosis in patients with coronary artery disease undergoing drug-eluting stenting. Coron. Artery Dis..

[21] Kurtul A., Yarlioglues M., Murat S.N., Celik I.E., Demircelik M.B., Ocek A.H., Duran M., Ergun G., Cetin M., Ornek E. (2015). Predictors of Chronic Total Occlusion in Nonculprit Artery in Patients with Acute Coronary Syndrome: Mean Platelet Volume and Uric Acid. Angiology.

[22] Ayoub M., Mashayekhi K., Behnes M., Schupp T., Akin M., Forner J., Akin I., Neumann F.-J., Westermann D., Rudolph V. (2023). Prognostic Value of Different Levels of Uric Acid in Patients with Coronary Chronic Total Occlusion Undergoing Percutaneous Coronary Intervention. J. Clin. Med..

[23] Oğuz N., Kırça M., Çetin A., Yeşilkaya A. (2017). Effect of uric acid on inflammatory COX-2 and ROS pathways in vascular smooth muscle cells. J. Recept. Signal Transduct..

[24] Higgins P., Dawson J., Lees K.R., McArthur K., Quinn T.J., Walters M.R. (2012). Xanthine Oxidase Inhibition For The Treatment Of Cardiovascular Disease: A Systematic Review and Meta-Analysis. Cardiovasc. Ther..

[25] Kanbay M., Huddam B., Azak A., Solak Y., Kadioglu G.K., Kirbas I., Duranay M., Covic A., Johnson R.J. (2011). A Randomized Study of Allopurinol on Endothelial Function and Estimated Glomular Filtration Rate in Asymptomatic Hyperuricemic Subjects with Normal Renal Function. Clin. J. Am. Soc. Nephrol..

[26] Kojima S., Matsui K., Hiramitsu S., Hisatome I., Waki M., Uchiyama K., Yokota N., Tokutake E., Wakasa Y., Jinnouchi H. (2019). Febuxostat for Cerebral and CaRdiorenovascular Events PrEvEntion StuDy. Eur. Heart J..

[27] Valeriani E., Pannunzio A., Palumbo I.M., Bartimoccia S., Cammisotto V., Castellani V., Porfidia A., Pignatelli P., Violi F. (2024). Risk of venous thromboembolism and arterial events in patients with hypoalbuminemia: A comprehensive meta-analysis of more than 2 million patients. J. Thromb. Haemost..

[28] Elrashidy M.H., Hassan M.H., Abass N.M. (2022). Uric Acid-to-Albumin Ratio as a Non-Invasive predictor for the Severity of Coronary Atherosclerosis. SVU-Int. J. Med. Sci..

[29] Tong J., Wang T., Wei Q., Hao Q., Yu F., Xu X., Zhen P. (2025). Serum Albumin is Linearly and Negatively Associated With the Risk of All-cause and Cardiovascular Death in Coronary Heart Disease Patients. Rev. Cardiovasc. Med..

[30] Trimarchi G., Pizzino F., Lilli A., De Caterina A.R., Esposito A., Dalmiani S., Mazzone A., Di Bella G., Berti S., Paradossi U. (2024). Advanced Lung Cancer Inflammation Index as Predictor of All-Cause Mortality in ST-Elevation Myocardial Infarction Patients Undergoing Primary Percutaneous Coronary Intervention. J. Clin. Med..

