# Supplementary material for: Prognostic Value of the Uric Acid-to-Albumin Ratio in Patients Undergoing Successful Percutaneous Coronary Intervention for Chronic Total Occlusion
Source: J Cardiovasc Dev Dis. 2026 Jun 22;13(6):282. doi: 10.3390/jcdd13060282 (PMC13301519; doi:10.3390/jcdd13060282)
Supplement: Supplementary file 1 [file jcdd-13-00282-s001.zip › jcdd-4344329-supplementary.pdf]

Table S1. Associations between UAR and MACCE and all-cause mortality in participants with CTO adjusted for all covariates in Model 3 except C-reactive protein.

	**Model 4**			
	MACCE		All-cause mortality	
	HR (95%CI)	*P*-value	HR (95%CI)	*P*-value
UAR (Per -1 unit)	1.06 (1.02–1.11)	0.001	1.10 (1.05–1.15)	<0.001
UAR (Tertiles)				
Low	Reference		Reference	
Medium	1.16 (0.75–1.78)	0.509	1.62 (0.74–3.54)	0.224
High	1.92 (1.26–2.93)	0.002	3.39 (1.62–7.09)	0.001
P for trend		0.002		0.001

Adjust for: DM, sex, age, HTN, eGFR, LVEF, Prior MI, Smoker, Multi-vessel disease, Statin, DAPT, LDL, BNP. eGFR, estimated Glomerular Filtration Rate; LVEF, Left Ventricular Ejection Fraction; CRP,C-reactive protein; LDL, low-density lipoprotein cholesterol; BNP brain natriuretic peptide; UAR, Uric Acid-to-Albumin Ratio; MACCE, Major Adverse Cardiovascular Events; HTN, Hypertension; DM, Diabetes Mellitus; Prior MI, Prior Myocardial Infarction; DAPT, Dual Antiplatelet Therapy.
